# Ischemic and Bleeding Events of Ticagrelor Monotherapy in Korean Patients With and Without Diabetes Mellitus: Insights From the TICO Trial

**DOI:** 10.3389/fphar.2020.620906

**Published:** 2021-01-14

**Authors:** Kyeong Ho Yun, Jae Young Cho, Seung Yul Lee, Sang Jae Rhee, Byeong Keuk Kim, Myeong Ki Hong, Yangsoo Jang, Seok Kyu Oh, the TICO Investigators

**Affiliations:** ^1^ Departments of Cardiovascular Medicine, Regional Cardiocerebrovascular Center, Wonkwang University Hospital, Iksan, South Korea; ^2^ Severance Cardiovascular Hospital, Yonsei University College of Medicine, Seoul, South Korea

**Keywords:** dual anti platelet therapy, diabetes mellitus, ticagrelor, aspirin, Prognosis, ischemia

## Abstract

**Background**: Ticagrelor monotherapy after 3 months dual antiplatelet therapy (DAPT) with aspirin and ticagrelor can reduce bleeding without increasing ischemic events after percutaneous coronary intervention (PCI). However, the impact of this approach among the patient with diabetes remains unknown.

**Methods**: This was a sub-analysis of the Ticagrelor Monotherapy after 3 months in the Patients Treated with New Generation Sirolimus Eluting Stent for Acute Coronary Syndrome (TICO) trial. After successful PCI, the patients were randomly assigned to ticagrelor monotherapy after 3-months DPAT or to ticagrelor-based 12-months DAPT. We compared ischemic events and bleeding events between the patients with diabetes and without diabetes for 12 months. Ischemic events were defined as death, myocardial infarction, ischemic stroke, transient ischemic attack, stent thrombosis, and any revascularizations. Bleeding events were defined according to the Thrombolysis in Myocardial Infarction (TIMI) criteria and Bleeding Academic Research Consortium (BARC) definition.

**Results**: Between August 2015 and October 2018, 3,056 patients were enrolled in the TICO trial, of which 835 (27.3%) had diabetes mellitus. Diabetes mellitus was associated with all evaluated ischemic and bleeding events. No significant differences in any ischemic events were observed in patients with diabetes between ticagrelor monotherapy after 3-months DAPT and ticagrelor-based 12-months DAPT (hazard ratio [HR] 0.83, 95% confidence interval [CI] 0.45–1.52, p = 0.540). In patients with diabetes, the overall incidence of bleeding complications during the 12-months follow-up period did not differ between the two treatment groups (HR 0.83, 95% CI 1.48–1.43, p = 0.505). However, ticagrelor monotherapy was significantly reduced both any TIMI bleeding and BARC three or five bleeding events in diabetes patients in the 3-months landmark analysis, after 3-months DAPT period (HR 0.20, 95% CI 0.07–0.59, p = 0.003).

**Conclusion**: In diabetic patients, ticagrelor monotherapy showed a lower incidence of bleeding complications after 3-months DAPT period, without increasing ischemic complications, compared with ticagrelor-based 12-months DAPT (ClinicalTrials.gov Identifier: NCT02494895).

## Introduction

Dual antiplatelet therapy (DAPT) with aspirin and a P2Y12 inhibitor for 12 months is a standard therapy in patients with acute coronary syndrome (ACS) underwent drug-eluting stent (DES) implantation (Levine et al., 2016; Valgimigli et al., 2018). The improved clinical performances of DES has reduced the risk of stent thrombosis; however, potent antiplatelet agents can increase the risk of bleeding complications. Therefore, determining the optimal duration of DAPT is especially important to prevent both ischemic and bleeding complications (Capodanno et al., 2015). Recently, short duration of DAPT, followed by P2Y12 inhibitor monotherapy, was shown to provide an optimal risk-benefit balance (Vranckx et al., 2018; Mehran et al., 2019; Kim et al., 2020). Ticagrelor Monotherapy after 3 months in the Patients Treated with New Generation Sirolimus Eluting Stent for Acute Coronary Syndrome (TICO) trial demonstrated that ticagrelor monotherapy after 3-months DAPT showed 34% reduction of net adverse events compared with 12-months DAPT with aspirin and ticagrelor, mainly reduction of bleeding events (Kim et al., 2020). However, additional studies remain necessary to evaluate several specific subgroups that have high ischemic or bleeding risks.

Diabetes mellitus is an important risk factor of both ischemic and bleeding complications. Pooled randomized trials showed diabetes was an independent predictor of major adverse cardiac events, cardiac death, and myocardial infarction (MI) after PCI (Kedhi et al., 2014). In the Platelet Inhibition and Patient Outcomes (PLATO) study, diabetic patients with ACS had a higher bleeding risk than non-diabetic patients in both clopidogrel and ticagrelor group (James et al., 2010). Therefore, the effect of antiplatelet strategy on the ischemic and bleeding events may differ between the patients with diabetes and without diabetes after PCI. This study investigated ischemic and bleeding events in the TICO trial according to diabetes mellitus status. We evaluated the impact of diabetes on the effect of two antiplatelet strategy, ticagroler monotherapy after 3-months DAPT and 12-months DAPT with aspirin and ticagrelor.

## Methods

### Study Population and Design

This is a sub-study of the TICO trial (clinicaltrials.gov identifier: NCT02494895). TICO trial was an investigator-initiated, multicenter, randomized, open-label trial conducted across 38 centers in South Korea. The rationale and detailed design have been previously published (Kim et al., 2019). In brief, total 3,056 patients with acute coronary syndrome (ACS) were randomly assigned to ticagrelor monotherapy after 3-months DPAT or to ticagrelor-based 12-months DAPT after successful percutaneous coronary intervention (PCI) with ultrathin bioresorbable polymer sirolimus-eluting stents (Orsiro; Biotronik AG). All DAPT treatment composed a combination of aspirin and ticagrelor. Randomization sequence was computer generated and stratified according to diabetes status and ST-elevation myocardial infarction (MI). Key exclusion criteria included the increased risk of bleeding associated with prior hemorrhagic stroke, traumatic brain injury or brain surgery within the past 6 months, internal bleeding within the past 6 weeks, need of oral anticoagulation therapy, and anemia (hemoglobin ≤8 g/dl).

After a 3-months DAPT treatment consisting of ticagrelor and aspirin, the aspirin was discontinued for the ticagrelor monotherapy group and continued in the 12-months DAPT group. The collection of each participant's current medication and adverse or end point-related events is performed at baseline and during 3, 6, 9, and 12 months of follow-up after PCI. Patients were categorized as diabetic if they received active treatment with insulin, an oral antidiabetic medication, or were treated through diet management.

### Endpoints

The outcomes of present study included any ischemic and bleeding complications within 12 months of PCI. The TICO trial defined major adverse cardiac and cerebrovascular events as death, MI, stent thrombosis, stroke, or target vessel revascularization (TVR). Additionally, transient ischemic attack, ischemic stroke, and non-TVR were analyzed as ischemic complications in this study according to Academic Research Consortium (Lansky et al., 2017; Garcia-Garcia et al., 2018). Bleeding complications were defined according to the Thrombolysis in Myocardial Infarction (TIMI) criteria and the Bleeding Academic Research Consortium (BARC) definition (Mehran et al., 2011). Endpoint detection and adjudication were obtained as previously outlined (Kim et al., 2020).

### Statistical Analyses

Patient characteristics were compared by diabetes status using independent Student's *t*-test or the Mann–Whitney *U* test in continuous variables and using a *χ* ^2^ test or Fisher's exact test in categorical variables. Kaplan–Meier estimates and log-rank test were used to determine the cumulative incidences of the ischemic and bleeding events. Hazard ratio (HR) and 95% confidence interval (CI) were generated, using Cox proportional hazards models. To determine the effect of diabetes on the outcomes, following covariates were used for adjusting: age, gender, body mass index, hypertension, dyslipidemia, and ejection fraction. Patients without endpoints between randomization and 12 months were censored at the time of death, last contact date in cases with lost to follow-up or withdrew consent, or 365 days, whichever came first. Treatment effects were estimated according to presence or absence of diabetes with interaction terms in a Cox proportional hazard model. Because the same treatment was given to both groups during the first 3 months, pre-specified 3-months landmark analyses were performed, after excluding the patients who experienced adverse events within this period. Analyses of both ischemic and bleeding events were performed using the intension-to-treat cohort. All statistical analyses were performed using R version 4.0.2. (The R foundation for Statistical Computing, Vienna, Austria). All *p*-values are two-sided, and a *p*-value lower than 0.05 was regarded as statistically significant.

## Results

**FIGURE 1:**
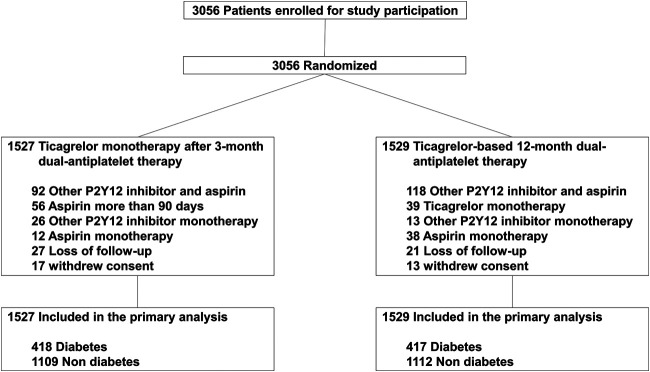
Participant flow in the present study.

Between August 2015 and October 2018, 3,056 patients were enrolled in the TICO trial, of which 835 (27.3%) had diabetes mellitus. Among diabetic patients, 82 (9.8%) patients were treated with insulin. The drug adherence and cross-over rates were similar between the two treatment strategy groups (Figure 1). Table 1 presents the baseline characteristics of patients, according to diabetic status. Diabetic patients presented worse clinical characteristics than nondiabetic patients. Patients with diabetes were older, more likely to report being current smokers, and had higher incidences of hypertension, dyslipidemia, and multivessel disease than nondiabetic patients. Medications other than aspirin, and procedures were well matched between the randomized treatment groups.

**TABLE 1:** Baseline clinical characteristics.

	Diabetes (*n* = 835)	No diabetes (*n* = 2,221)	*p* value
Age (years)	63.3 ± 10.5	60.0 ± 10.7	<0.001
Male (%)	612 (73.3)	1816 (81.8)	<0.001
Body mass index (kg/m^2^)	25.1 ± 3.4	24.9 ± 3.2	0.154
Hypertension (%)	570 (68.3)	971 (43.7)	<0.001
Dyslipidemia (%)	535 (64.1)	1,311 (59.0)	0.011
Current smoker (%)	266 (31.9)	876 (39.4)	<0.001
Previous stroke (%)	62 (7.4)	64 (2.9)	<0.001
Myocardial infarction (%)	534 (64.0)	1,596 (71.9)	<0.001
Ejection fraction (%)	53.9 ± 12.6	54.8 ± 11.8	0.082
Hemoglobin A1c (%)	7.7 ± 1.6	5.9 ± 1.0	<0.001
eGFR (mL/min/1.73m^2^)	71.2 ± 25.8	78.8 ± 22.2	<0.001
12-months DPAT group	417 (49.9)	1,112 (50.1)	0.950
Angiographic and procedural findings			
Culprit lesion (%)			0.093
Left main	27 (3.2)	53 (2.4)	
Left anterior descending	399 (47.8)	1,129 (50.8)	
Left circumflex	130 (15.6)	380 (17.1)	
Right coronary artery	279 (33.4)	659 (29.7)	
Multi-vessel disease (%)	536 (64.2)	1,167 (52.5)	<0.001
Femoral approach (%)	369 (44.2)	989 (44.5)	0.867
Stent number per patient	1.42 ± 0.72	1.35 ± 0.65	0.023
Stent diameter (mm)	3.10 ± 0.45	3.16 ± 0.45	0.001
Total stent length (mm)	36.8 ± 21.7	34.0 ± 20.1	0.001
Discharge medication			
ACEI/ARB (%)	570 (68.3)	1,449 (65.2)	0.116
Beta blocker (%)	548 (65.6)	1,468 (66.1)	0.808
CCB (%)	114 (13.7)	283 (12.7)	0.505
Statin (%)	819 (98.1)	2,174 (97.9)	0.729

DAPT, dual antiplatelet therapy; eGFR, estimated glomerular filtration rate; ACEI, angiotensin converting enzyme inhibitor; ARB, angiotensin receptor blocker; CCB, calcium channel blocker.

**FIGURE 2:**
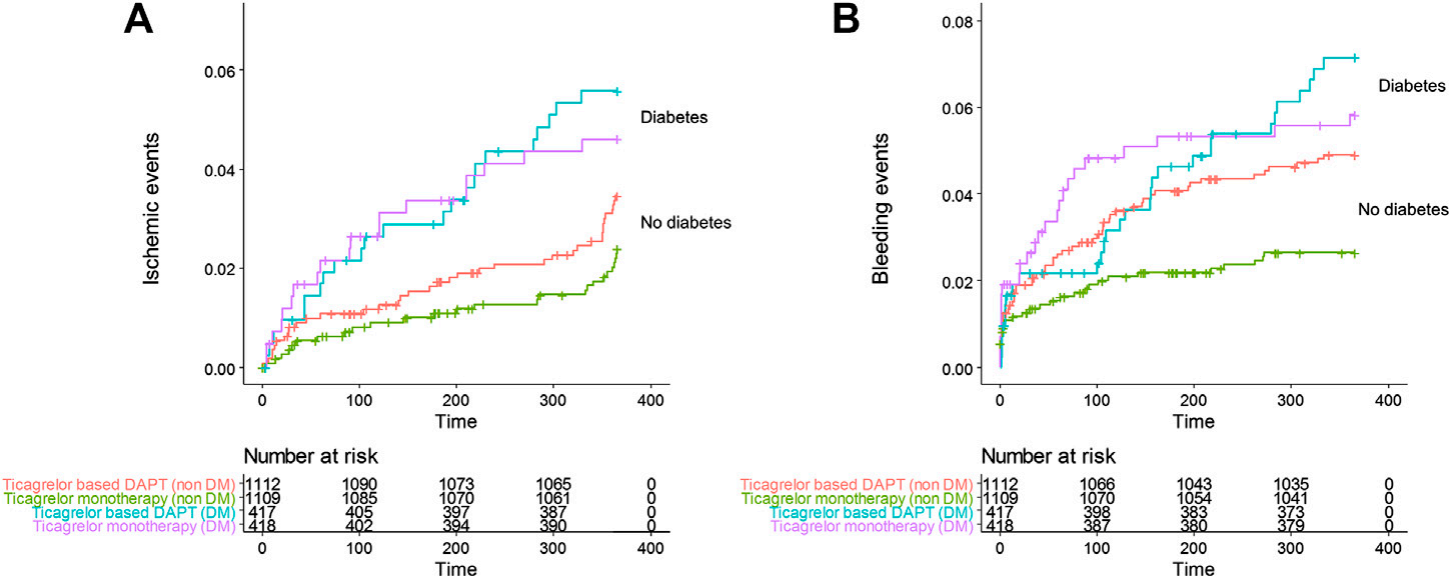
Cumulative incidence of **(A)** all ischemic events including death, MI, stent thrombosis, transient ischemic attack, stroke, and any revascularization, **(B)** all Thrombolysis in Myocardial Infarction bleeding in the ticagrelor based 12-months dual antiplatelet group and ticagrelor monotherapy group in patients with diabetes and no diabetes.

The effect of diabetes mellitus on all evaluated ischemic and bleeding events showed in Table 2 and Figure 2. Diabetic patients experienced a higher incidence of bleeding complications (6.3% vs. 3.7%, *p* = 0.002). Additionally, diabetic patients showed a higher incidence of all ischemic events, extended definitions in this study, than nondiabetic patients (5.0% vs. 2.9%, *p* = 0.004). No significant difference was found in any ischemic or bleeding events according to insulin treatment or baseline HbA1c levels (Table 3).

**TABLE 2:** Association of diabetes status with ischemic and bleeding events.

	Diabetes (*n* = 835)	No diabetes (*n* = 2,221)	HR (95% CI)	*p* value
Ischemic events (%)				
Death	22 (2.6)	17 (0.8)	3.47 (1.85–6.54)	<0.001
Myocardial infarction	10 (1.2)	7 (0.3)	3.87 (1.47–10.16)	0.006
Stent thrombosis	7 (0.8)	3 (0.1)	6.24 (1.61–24.14)	0.008
MACE^a^	34 (4.1)	48 (2.2)	1.91 (1.23–2.96)	0.004
Bleeding events (%)				
TIMI major bleeding	30 (3.6)	40 (1.8)	2.02 (1.26–3.25)	0.004
TIMI minor bleeding	23 (2.8)	43 (1.9)	1.44 (0.87–2.39)	0.159
All TIMI bleeding	53 (6.3)	83 (3.7)	1.73 (1.22–2.44)	0.002

HR, hazard ratio; CI, confidence interval; TIMI, Thrombolysis in Myocardial Infarction.

^a^ Major adverse cardiac and cerebrovascular events indicated composite of death, myocardial infarction, stent thrombosis, stroke, and target vessel revascularization.

**TABLE 3:** Outcomes in patients with diabetes.

	Ticagelor monotherapy	Ticagrelor-based DAPT	HR (95%CI)	*p* value	*p* value (interaction)
All-cause mortality					
Insulin	0/42 (0.0)	4/40 (10.0)		0.052^a^	0.996
No insulin	8/376 (2.1)	10/377 (2.7)	0.81 (0.32–2.04)	0.649	
HbA1c >7.0%	3/159 (1.9)	6/166 (3.6)	0.52 (0.13–2.08)	0.356	0.996
HbA1c ≤ 7.0%	0/132 (0.0)	0/121 (0.0)		1.000^a^	
Major adverse cardiac and cerebrovascular events					
Insulin	0/42 (0.0)	5/40 (12.5)		0.024^a^	0.997
No insulin	13/376 (3.5)	16/377 (4.2)	0.82 (0.40–1.71)	0.599	
HbA1c >7.0%	4/159 (2.5)	8/166 (4.8)	0.52 (0.16–1.72)	0.283	0.997
HbA1c ≤ 7.0%	3/132 (2.3)	1/121 (0.8)	2.8 (0.29–26.95)	0.372	
Any ischemic events					
Insulin	1/42 (2.4)	6/40 (15.0)	0.14 (0.02–1.19)	0.072	0.075
No insulin	18/376 (4.8)	17/377 (4.5)	1.08 (0.56–2.09)	0.827	
HbA1c >7.0%	8/159 (5.0)	8/166 (4.8)	1.04 (0.39–2.78)	0.933	0.075
HbA1c ≤ 7.0%	5/132 (3.8)	1/121 (0.8)	4.7 (0.55–40.25)	0.158	
TIMI major bleeding					
Insulin	1/42 (2.4)	2/40 (5.0)	0.44 (0.04–4.84)	0.501	0.730
No insulin	11/376 (2.9)	16/377 (4.2)	0.70 (0.32–1.51)	0.361	
HbA1c >7.0%	7/159 (4.4)	5/166 (3.0)	1.49 (0.47–4.70)	0.494	0.730
HbA1c ≤ 7.0%	1/132 (0.8)	4/121 (3.3)	0.23 (0.03–2.04)	0.186	
All TIMI bleeding					
Insulin	3/42 (7.1)	6/40 (15.0)	0.44 (0.11–1.77)	0.247	0.341
No insulin	21/376 (5.6)	23/377 (6.1)	0.93 (0.52–1.68)	0.810	
HbA1c >7.0%	14/159 (8.8)	9/166 (5.4)	1.68 (0.73–3.89)	0.222	0.341
HbA1c ≤ 7.0%	2/132 (1.5)	8/121 (6.6)	0.23 (0.05–1.06)	0.059	

Major adverse cardiac and cerebrovascular events (MACE) indicated composite of death, myocardial infarction, stent thrombosis, stroke, and target vessel revascularization (TVR). Any ischemic events indicated transient ischemic attack and non-TVR in addition to MACE.

^a^ Fisher's exact test.

**FIGURE 3:**
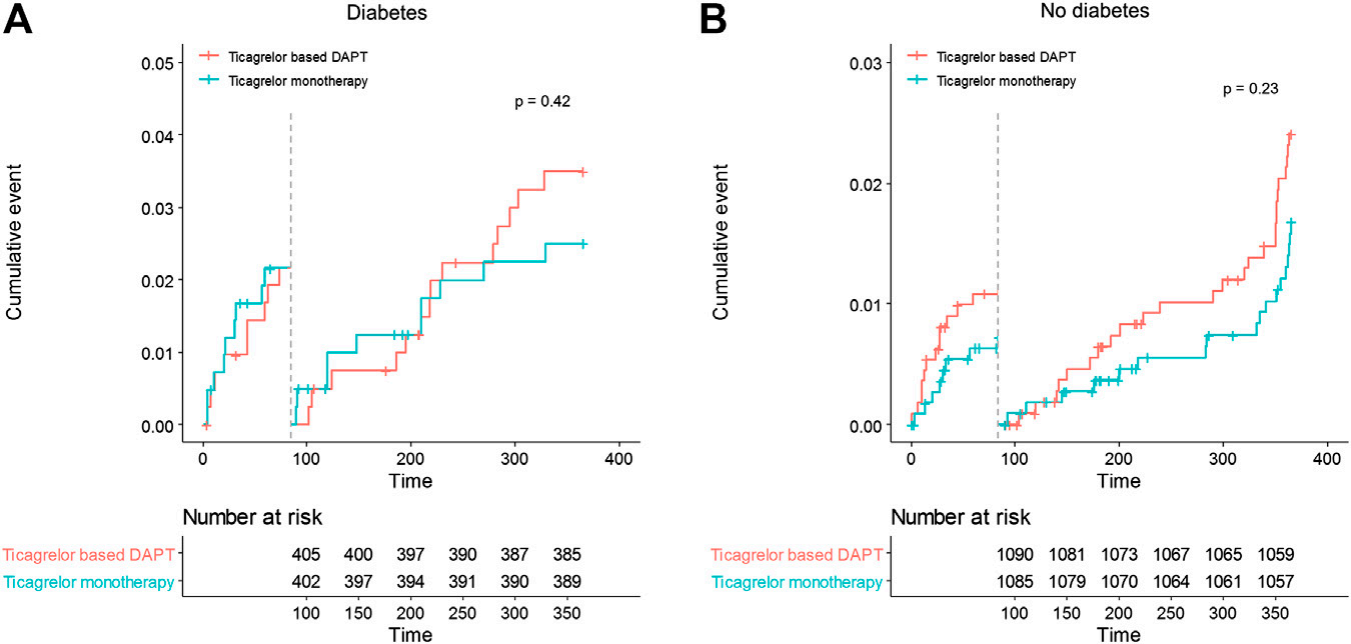
Landmark analysis of ischemic events at 3 months. Between 3 and 12 months, the hazard ratio of ticagrelor monotherapy was 0.72 (95% CI 0.32–1.61) in diabetes, 0.69 (0.38–1.26) in no diabetes.

No significant differences in any ischemic events were observed in patients with diabetes between ticagrelor monotherapy after 3-months DAPT and ticagrelor-based 12-months DAPT (HR 0.83, 95% CI 0.45–1.52, *p* = 0.540) (Table 4). The incidence of death, MI, stroke, and any revascularizations was similar between ticagrelor monotherapy after 3-months DAPT and ticagrelor-based 12-months DAPT in both patients with diabetes and without diabetes. The 3-months landmark analysis showed similar incidence of ischemic complications during 3–12 months between the two treatment strategies (Figure 3). In the overall trial population, there was no significant interaction between diabetes status and treatment group with respect to ischemic events.

**TABLE 4:** Ischemic events according to treatment group in patients with and without diabetes.

	Ticagelor monotherapy	Ticagrelor-based DAPT	HR (95%CI)	*p* value	*p* value (interaction)
Death					
Diabetes	8/418 (1.9)	14/417 (3.4)	0.57 (0.24–1.36)	0.204	0.491
No diabetes	8/1,109 (0.7)	9/1,112 (0.8)	0.90 (0.35–2.32)	0.819	
Cardiac death					
Diabetes	5/418 (1.2)	8/417 (1.9)	0.62 (0.20–1.91)	0.408	0.835
No diabetes	2/1,109 (0.2)	4/1,112 (0.4)	0.50 (0.09–2.74)	0.426	
Myocardial infarction					
Diabetes	5/418 (1.2)	5/417 (1.2)	1.0 (0.29–3.46)	0.997	0.154
No diabetes	1/1,109 (0.1)	6/1,112 (0.5)	0.17 (0.02–1.39)	0.098	
Stent thrombosis					
Diabetes	4/418 (1.0)	3/417 (0.7)	1.33 (0.30–5.93)	0.710	0.774
No diabetes	2/1,109 (0.2)	1/1,112 (0.1)	2.0 (0.18–22.14)	0.569	
TIA					
Diabetes	3/418 (0.7)	2/417 (0.5)	1.50 (0.25–8.95)	0.659	0.998
No diabetes	0/1,109 (0.0)	3/1,112 (0.3)		0.250^a^	
Ischemic stroke					
Diabetes	0/418 (0.0)	1/417 (0.2)		0.499^a^	0.998
No diabetes	5/1,109 (0.5)	8/1,112 (0.7)	0.63 (0.21–1.92)	0.414	
TVR					
Diabetes	2/418 (0.5)	2/417 (0.5)	0.99 (0.14–7.05)	0.994	0.812
No diabetes	6/1,109 (0.5)	8/1,112 (0.7)	0.76 (0.26–2.18)	0.604	
Non-TVR					
Diabetes	3/418 (0.7)	0/417 (0.0)		0.249^a^	0.997
No diabetes	9/1,109 (0.8)	6/1,112 (0.5)	1.51 (0.54–4.23)	0.438	
Any revascularization					
Diabetes	5/418 (1.2)	2/417 (0.5)	2.50 (0.49–12.88)	0.274	0.288
No diabetes	13/1,109 (1.2)	14/1,112 (1.3)	0.94 (0.44–1.99)	0.862	
Any ischemic events					
Diabetes	19/418 (4.5)	23/417 (5.5)	0.83 (0.45–1.52)	0.540	0.645
No diabetes	26/1,109 (2.3)	38/1,112 (3.4)	0.69 (0.42–1.13)	0.138	

TIA, transient ischemic attack; TVR, target vessel revascularization.

^a^ Fisher's exact test.

**FIGURE 4:**
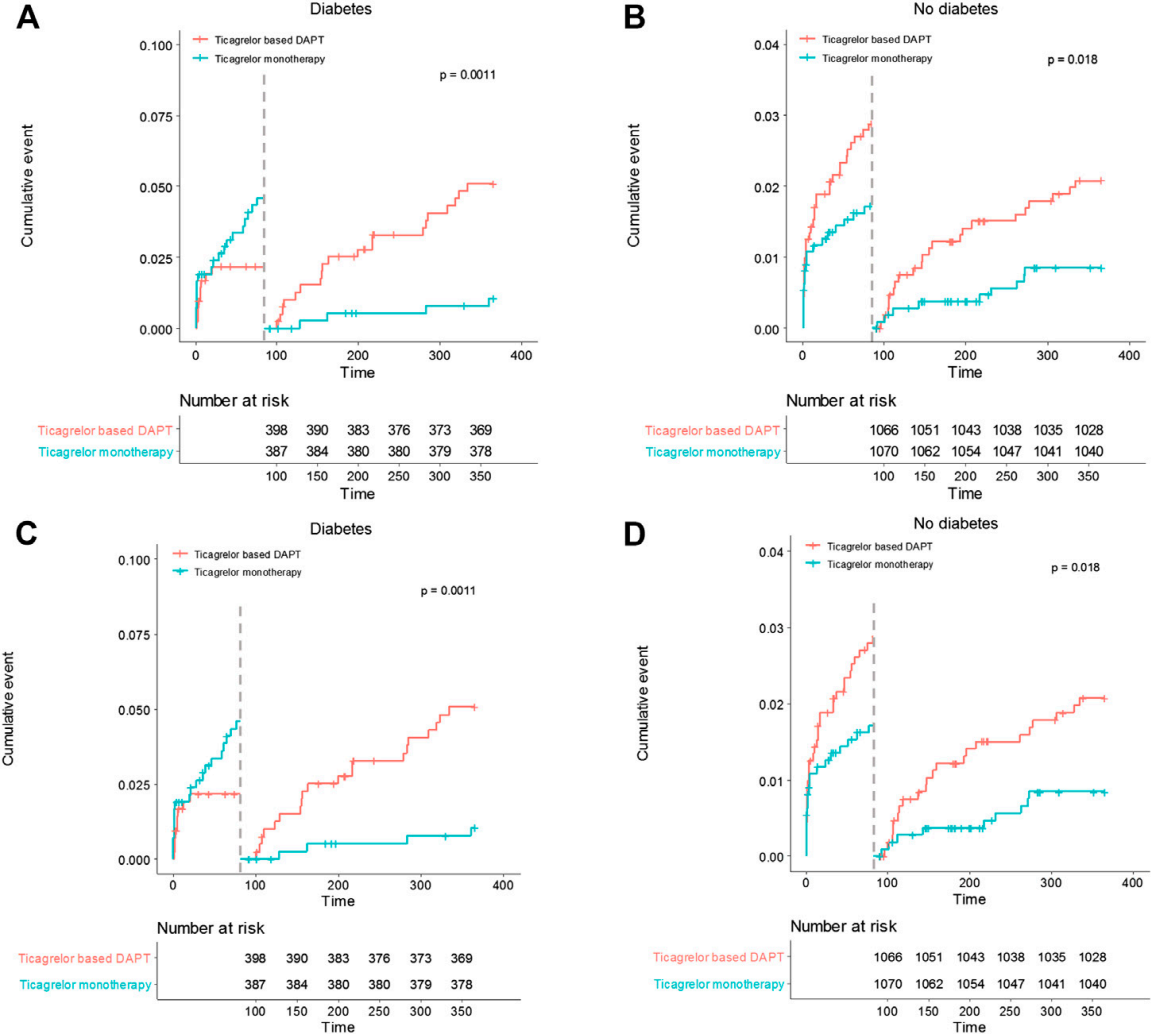
Landmark analysis of bleeding events at 3 months. **(A)** and **(B)** demonstrated TIMI major or minor bleeding events. Between 3 and 12 months, the hazard ratio of ticagrelor monotherapy was 0.20 (95% CI 0.07–0.59) in diabetes, 0.41 (0.19–0.88) in no diabetes. **(C)** and **(D)** demonstrated BARC 3 or 5 bleeding events. The hazard ratio of ticagrelor monotherapy was 0.20 (95% CI 0.07–0.59) in diabetes, 0.41 (0.19–0.88) in no diabetes between 3 and 12 months.

In diabetic patients, the overall incidences of bleeding events during the 12-months follow-up period did not differ between the two treatment groups (HR 0.83, 95% CI 1.48–1.43, *p* = 0.505) (Table 5). However, ticagrelor-based 12-months DAPT increased bleeding complications in the 3-months landmark analysis in both patients with diabetes and without diabetes (log-rank *p* = 0.0011 in patients with diabetes; *p* = 0.018 in patients without diabetes, Figure 4). Ticagrelor monotherapy was significantly reduced both TIMI major or minor bleeding events and BARC 3 or 5 bleeding events in diabetes patients after 3-months DAPT period compared with 12-months DAPT (HR 0.20, 95% CI 0.07–0.59, *p* = 0.003 by Cox proportional hazed regression analysis) (Figures 4A,C). In the overall population, interaction testing between diabetes status and treatment group for bleeding events was non-significant.

**TABLE 5:** Bleeding events according to treatment group in patients with and without diabetes.

	Ticagelor monotherapy	Ticagrelor-based DAPT	HR (95%CI)	*p* value	*p* value (interaction)
Fatal bleeding					
Diabetes	0/418 (0.0)	1/417 (0.2)		0.499^a^	1.000
No diabetes	0/1,109 (0.0)	1/1,112 (0.1)		1.000^a^	
BARC 3A bleeding					
Diabetes	10/418 (2.4)	9/417 (2.2)	1.11 (0.45–2.74)	0.818	0.244
No diabetes	15/1,109 (1.4)	26/1,112 (2.3)	0.58 (0.31–1.09)	0.090	
BARC 3B bleeding					
Diabetes	12/418 (2.9)	17/417 (4.1)	0.71 (0.34–1.49)	0.364	0.489
No diabetes	13/1,109 (1.2)	26/1,112 (2.3)	0.5 (0.26–0.97)	0.041	
BARC 3C bleeding					
Diabetes	2/418 (0.5)	2/417 (0.5)	0.99 (0.14–7.03)	0.993	0.993
No diabetes	1/1,109 (0.1)	1/1,112 (0.1)	1.0 (0.06–16.06)	0.997	
BARC 3 or 5 bleeding					
Diabetes	24/418 (5.7)	29/417 (7.0)	0.83 (0.48–1.43)	0.505	0.219
No diabetes	29/1,109 (0.6)	54/1,112 (4.9)	0.54 (0.34–0.84)	0.007	
TIMI major bleeding					
Diabetes	12/418 (2.9)	18/417 (4.3)	0.67 (0.32–1.39)	0.281	0.513
No diabetes	13/1,109 (1.2)	27/1,112 (2.4)	0.48 (0.25–0.94)	0.031	
TIMI minor bleeding					
Diabetes	12/418 (2.9)	11/417 (2.6)	1.09 (0.48–2.47)	0.838	0.245
No diabetes	16/1,109 (1.4)	27/1,112 (2.4)	0.59 (0.32–1.10)	0.098	
All TIMI bleeding					
Diabetes	24/418 (5.7)	29/417 (7.0)	0.83 (0.48–1.43)	0.505	0.219
No diabetes	29/1,109 (0.6)	54/1,112 (4.9)	0.54 (0.34–0.84)	0.007	

BARC, Bleeding Academic Research Consortium; TIMI, Thrombolysis in Myocardial Infarction.

^a^ Fisher's exact test.

## Discussion

The key findings from this TICO sub-analysis include (1) ticagrelor-based 12-months DAPT did not reduced ischemic events in patients with diabetes compared with ticagrelor monotherapy after 3 months of DAPT, (2) ticagrelor monotherapy showed 80% reduction in the incidence of both any TIMI bleeding and BARC three or five bleeding after 3-months DAPT period in the patients with diabetes, and (3) the effect of ticagrelor monotherapy after 12-months DAPT on ischemic or bleeding events was not affected by diabetes status. Actually, ticagrelor monotherapy showed lower incidence of bleeding complications without increasing ischemic complications during 3–12 months irrespective of presence or absence of diabetes mellitus.

Because bleeding risks increase with prolonged DAPT, several studies have explored methods to shorten DAPT duration. The 6-months vs. 12-months or Longer Dual Antiplatelet Therapy After Percutaneous Coronary Intervention in Patients With Acute Coronary Syndrome (SMART-DATE) trial reported that short DAPT less than 6 months showed increased risk of MI compared with 12-months DAPT in patients with ACS (Hahn et al., 2018). The TICO trial tested a strategy in which aspirin use was discontinued while continuing ticagrelor in patients with ACS (Kim et al., 2020). As a result, ticagrelor monotherapy after 3-months of DAPT significantly reduced composite outcomes compared with ticagrelor-based 12-months DAPT, reducing the risk of major bleeding, without increasing the risk of major adverse cardio-cerebrovascular events. However, studies about the effects of P2Y12 monotherapy on high risk patients such as diabetes, chronic kidney disease, or underwent complex intervention, and studies using various endpoint including minor events remains uncertain.

Diabetes mellitus is a major risk factor of atherosclerosis, disease progression, and restenosis after DES implantation (The BARI investigators, 1997; Van Belle et al., 2002; Bangalore et al., 2012). In diabetic patients, increased platelet activity and adhesion have been observed, resulting in a higher incidence of cardiovascular events and the reduced antithrombotic efficacy of treatments based on aspirin and clopidogrel, compared with nondiabetic patients (Jung et al., 2015; Santilli et al., 2015). A meta-analysis showed that, although prolonged DAPT had no significant impacts on the rates of mortality, MI, and stoke, short DAPT (<6 months) followed by aspirin monotherapy was associated with a significant increase in the rate of stent thrombosis in diabetic patients compared with nondiabetic patients (Gargiulo et al., 2016; Sharma et al., 2018). Because of the prothrombotic state associated with diabetes, the evaluation of the efficacy of antiplatelet monotherapy after 3-months DAPT compared to 12-months DAPT with aspirin and a P2Y12 inhibitor for among patients with diabetes is of major importance. Actually, diabetes is considered to an indicator that a patient will benefit from prolonged DAPT strategy when calculated DAPT scores (Yeh et al., 2016). Recent evidence has indicated that aspirin therapy is insufficient to prevent newly developed serious cardiovascular events and may, instead, cause major bleeding events during the primary prevention era (ASCEND Study Collaborative Group, 2018). Therefore, aspirin alone is considered an insufficient treatment for ischemic events in diabetic patients.

The present study demonstrated that ticagrelor monotherapy did not increase ischemic events including stent thrombosis, and was not associated with bleeding risks in diabetic patients. To date, three randomized trials have examined ticagrelor monotherapy (Vranckx et al., 2018; Mehran et al., 2019; Kim et al., 2020). Although the study design and endpoints were different, GLOBAL LEADERS trial and Ticagrelor with Aspirin or Alone in High-Risk Patients after Coronary Intervention (TWILIGHT) trial both showed similar incidences of major cardiovascular events between the ticagrelor monotherapy group and the DAPT group among diabetic patients. The sub-analysis of TWILIGHT trial demonstrated that ticagrelor monotherapy showed 66% reduction of BARC 3 or 5 bleeding without increased risk of ischemic events (Angiolillo et al., 2020). This TICO sub-analysis also demonstrated comparable risk reduction of both TIMI major or minor and BARC 3 or 5 bleeding without increased incidence of ischemic events in patients with diabetes, although we used more extended definition of ischemic complications than the original TICO study. Moreover, we showed that even among diabetic patients, the discontinuation of aspirin after short-duration DAPT could prevent bleeding complications.

This study has several limitations. First, this study represents a subanalysis and was not designed to test the efficacy or safety of ticagrelor monotherapy in diabetic patients. Second, the statistical power of the TICO trial was calculated by estimating the occurrence of a composite outcome. The comparisons of each individual outcome, especially adverse cardiovascular and cerebrovascular events, could be underpowered. Third, we did not investigate the incidence of minor bleeding events such as BARC 1 or 2 bleeding. TICO study designed to evaluate net clinical effects. Study committee discussed whether importance of each ischemic and bleeding events were similar, and decided to exclude minor bleeding events. Fourth, pathophysiologic parameters, such as platelet reactivity, were not measured. The incidence of high on-treatment platelet reactivity might be higher in diabetic patients than in nondiabetic patients. However, our randomization strategy stratified by diabetes status and ST-elevation MI. Finally, it should be cautious to apply the results of this study to non-Asians or patients receiving drug-eluting stents other than Orsiro stents.

In conclusion, ticagrelor monotherapy showed a lower incidence of bleeding complications after 3-months DAPT period, without increasing ischemic complications, compared with ticagrelor-based 12-months DAPT in diabetic patients.

## Data Availability Statement

The original contributions presented in the study are included in the article/Supplementary Material, further inquiries can be directed to the corresponding authors.

## Ethics Statement

The studies involving human participants were reviewed and approved by institutional review board at each hospital. The patients provided their written informed consent to participate in this study.

## Author Contributions

KY and JC study design and interpretation of results. SR, SO, B-KK, M-KH, and YJ data collection. KY, JC, and S-YL: data analysis. SR and B-KK validation. JC and SO supervision. KY preparation of manuscript. KY, JC, and S-YL manuscript review and editing. All authors contributed to the article and approved the submitted version.

## Conflict of Interest

The authors declare that the research was conducted in the absence of any commercial or financial relationships that could be construed as a potential conflict of interest.

## References

[B1] Angiolillo D. J. Baber U. Sartori S. Briguori C. Dangas G. Cohen D. J. (2020). Ticagrelor with or without aspirin in high-risk patients with diabetes mellitus undergoing percutaneous coronary intervention. J. Am. Coll. Cardiol. 75, 2403–2413. 10.1016/j.jacc.2020.03.008 32240760

[B2] Bangalore S. Kumar S. Fusaro M. Amoroso N. Kirtane A. J. Byrne R. A. (2012). Outcomes with various drug eluting or bare metal stents in patients with diabetes mellitus: mixed treatment comparison analysis of 22,844 patient years of follow-up from randomised trials. BMJ. 345, e5170, 10.1136/bmj.e5170 22885395 PMC3415955

[B3] ASCEND Study Collaborative Group Bowman L. Bowman L. Mafham M. Wallendszus K. Stevens W. Buck G. (2018). Effects of aspirin for primary prevention in persons with diabetes mellitus. N. Engl. J. Med. 379, 1529–1539. 10.1056/NEJMoa1804988 30146931

[B4] Capodanno D. Gargiulo G. Buccheri S. Giacoppo D. Capranzano P. Tamburino C. (2015). Meta-analyses of dual antiplatelet therapy following drug-eluting stent implantation: do bleeding and stent thrombosis weigh similar on mortality?. J. Am. Coll. Cardiol. 66, 1639–1640. 10.1016/j.jacc.2015.05.085 26429096

[B5] Garcia-Garcia H. M. McFadden E. P. Farb A. Mehran R. Stone G. W. Spertus J. (2018). Standardized end point definitions for coronary intervention trials: the academic research consortium-2 consensus document. Circulation. 137, 2635–2650. 10.1161/CIRCULATIONAHA.117.029289 29891620

[B6] Gargiulo G. Windecker S. da Costa B. R. Feres F. Hong M. K. Gilard M. (2016). Short term versus long term dual antiplatelet therapy after implantation of drug eluting stent in patients with or without diabetes: systematic review and meta-analysis of individual participant data from randomised trials. BMJ. 355, i5483 10.1136/bmj.i5483 27811064 PMC5094199

[B7] Hahn J. Y. Song Y. B. Oh J. H. Cho D. K. Lee J. B. Doh J. H. (2018). 6-month versus 12-month or longer dual antiplatelet therapy after percutaneous coronary intervention in patients with acute coronary syndrome (SMART-DATE): a randomised, open-label, non-inferiority trial. Lancet. 391, 1274–1284. 10.1016/S0140-6736(18)30493-8 29544699

[B8] James S. Angiolillo D. J. Cornel J. H. Erlinge D. Husted S. Kontny F. (2010). Ticagrelor vs. clopidogrel in patients with acute coronary syndromes and diabetes: a substudy from the PLATelet inhibition and patient Outcomes (PLATO) trial. Eur. Heart J. 31, 3006–3016. 10.1093/eurheartj/ehq325 20802246 PMC3001588

[B9] Jung J. H. Tantry U. S. Gurbel P. A. Jeong Y. H. (2015). Current antiplatelet treatment strategy in patients with diabetes mellitus. Diabetes Metab. J. 39, 95–113. 10.4093/dmj.2015.39.2.95 25922803 PMC4411553

[B10] Kedhi E. Généreux P. Palmerini T. McAndrew T. C. Parise H. Mehran R. (2014). Impact of coronary lesion complexity on drug-eluting stent outcomes in patients with and without diabetes mellitus: analysis from 18 pooled randomized trials. J. Am. Coll. Cardiol. 63, 2111–2118. 10.1016/j.jacc.2014.01.064 24632279

[B11] Kim B. K. Hong S. J. Cho Y. H. Yun K. H. Kim Y. H. Suh Y. (2020). Effect of ticagrelor monotherapy vs ticagrelor with aspirin on major bleeding and cardiovascular events in patients with acute coronary syndrome: the TICO randomized clinical trial. J. Am. Med. Assoc. 323, 2407–2416. 10.1001/jama.2020.7580 PMC7298605 32543684

[B12] Kim C. Hong S. J. Shin D. H. Kim B. K. Ahn C. M. Kim J. S. (2019). Randomized evaluation of ticagrelor monotherapy after 3-month dual-antiplatelet therapy in patients with acute coronary syndrome treated with new-generation sirolimus-eluting stents: TICO trial rationale and design. Am. Heart J. 212, 45–52. 10.1016/j.ahj.2019.02.015 30933857

[B13] Lansky A. J. Messé S. R. Brickman A. M. Dwyer M. van der Worp H. B. Lazar R. M. (2017). Proposed standardized neurological endpoints for cardiovascular clinical trials: an academic research Consortium initiative. J. Am. Coll. Cardiol. 69, 679–691. 10.1016/j.jacc.2016.11.045 28183511

[B14] Levine G. N. Bates E. R. Bittl J. A. Brindis R. G. Fihn S. D. Fleisher L. A. (2016). 2016 ACC/AHA guideline Focused Update on duration of Dual Antiplatelet therapy in patients with coronary artery disease: a report of the American college of cardiology/American Heart association Task Force on Clinical practice guidelines. J. Am. Coll. Cardiol. 68, 1082–1115. 10.1016/j.jacc.2016.03.513 27036918

[B15] Mehran R. Baber U. Sharma S. K. Cohen D. J. Angiolillo D. J. Briguori C. (2019). Ticagrelor with or without aspirin in high-risk patients after PCI. N. Engl. J. Med. 381, 2032–2042. 10.1056/NEJMoa1908419 31556978

[B16] Mehran R. Rao S. V. Bhatt D. L. Gibson C. M. Caixeta A. Eikelboom J. (2011). Standardized bleeding definitions for cardiovascular clinical trials: a consensus report from the Bleeding Academic Research Consortium. Circulation. 123, 2736–2747. 10.1161/CIRCULATIONAHA.110.009449 21670242

[B17] Santilli F. Simeone P. Liani R. Davì G. (2015). Platelets and diabetes mellitus. Prostag. Other Lipid Mediat. 120, 28–39. 10.1016/j.prostaglandins.2015.05.002 25986598

[B18] Sharma A. Garg A. Elmariah S. Drachman D. Obiagwu C. Vallakati A. (2018). Duration of dual antiplatelet therapy following drug-eluting stent implantation in diabetic and non-diabetic patients: a systematic review and meta-analysis of randomized controlled trials. Prog. Cardiovasc. Dis. 60, 500–507. 10.1016/j.pcad.2017.12.003 29277295

[B19] The BARI investigators(1997). Influence of diabetes on 5-year mortality and morbidity in a randomized trial comparing CABG and PTCA in patients with multivessel disease: the Bypass Angioplasty Revascularization Investigation (BARI). Circulation. 96, 1761–1769. 10.1161/01.cir.96.6.1761 9323059

[B20] Valgimigli M. Bueno H. Byrne R. A. Collet J. P. Costa F. Jeppsson A. (2018). 2017 ESC focused update on dual antiplatelet therapy in coronary artery disease developed in collaboration with EACTS: the Task Force for dual antiplatelet therapy in coronary artery disease of the European Society of Cardiology (ESC) and of the European Association for Cardio-Thoracic Surgery (EACTS). Eur. Heart J. 39, 213–260. 10.1093/eurheartj/ehx419 28886622

[B21] Van Belle E. Périé M. Braune D. Chmaït A. Meurice T. Abolmaali K. (2002). Effects of coronary stenting on vessel patency and long-term clinical outcome after percutaneous coronary revascularization in diabetic patients. J. Am. Coll. Cardiol. 40, 410–417. 10.1016/s0735-1097(02)01971-x 12142104

[B22] Vranckx P. Valgimigli M. Jüni P. Hamm C. Steg P. G. Heg D. (2018). Ticagrelor plus aspirin for 1 month, followed by ticagrelor monotherapy for 23 months vs aspirin plus clopidogrel or ticagrelor for 12 months, followed by aspirin monotherapy for 12 months after implantation of a drug-eluting stent: a multicentre, open-label, randomised superiority trial. Lancet. 392, 940–949. 10.1016/S0140-6736(18)31858-0 30166073

[B23] Yeh R. W. Secemsky E. A. Kereiakes D. J. Normand S. L. Gershlick A. H. Cohen D. J. (2016). Development and validation of a prediction rule for benefit and harm of dual antiplatelet therapy beyond 1 year after percutaneous coronary intervention. J. Am. Med. Assoc. 315, 1735–1749. 10.1001/jama.2016.3775 PMC5408574 27022822

